# Multiple Sclerosis and Secondary Psychosis: Case Report and Literature Review

**DOI:** 10.1192/bjo.2026.11878

**Published:** 2026-06-30

**Authors:** Nilofar Rahman, Nihal Fernando, Ankush Singhal

**Affiliations:** 1 Pennine care foundation trust, Tameside, United Kingdom; 2 Pennine care foundation trust, Oldham, United Kingdom

## Abstract

**Aims:**

Multiple sclerosis (MS) is the most common demyelinating neurological diseases worldwide. Around 2.3 million people globally and 100,000 people in the UK were living with MS. Psychotic presentation is rare, but it may affect 2-4% of the individuals with MS, which is more than in the general population (1%). The psychotic symptoms reported in multiple sclerosis included both affective and schizophrenia-like symptoms with a predominance of positive psychotic symptoms, most frequently persecutory delusions.

**Methods:**

Case reports

Patient X is a 35-year-old female of Asian origin, with an established diagnosis of Multiple sclerosis, presented to the crisis team with thoughts to end her life as she firmly believed with impregnable conviction that her husband was unfaithful and was having an affair with the patient’s mother for the last 3 months. It impacted her sleep and overall quality of life as she used to keep up throughout the night to gather evidence of her husband’s infidelity. She was diagnosed with ‘Secondary psychotic syndrome, with delusions’ according to ICD 11. (6E61.1) The diagnosis of Multiple sclerosis was established around two years prior to her psychotic presentation which was treated with Natalizumab infusion. During the time of her presentation to our service, the symptoms related to MS was stable with no significant findings on neurological examination. X was commenced on a low dose of antipsychotic, initially Quetiapine (which she declined to take due to metabolic side effect) and then on Aripiprazole, 10 mg once daily. X showed considerable improvement within four weeks of initiation of the antipsychotic along with psychosocial support. She has been followed up by local neurology specialist teams actively and remained stable with respect to the demyelinating process secondary to MS on repeat MRI.

**Results:**

**Discussion:**

Psychotic symptoms may develop during the onset of MS or more frequently during illness as seen in this case. Regarding management, presence of Multiple sclerosis also makes the individual vulnerable to the side effects of antipsychotic drug like extrapyramidal side effects. Psychotic symptoms can be secondary to the demyelination process or results as a side effect of medication to treat MS like corticosteroids and beta- interferon.

**Conclusion:**

Although, cognitive and affective symptoms like depression are very common in pertinent among patients with MS, psychotic symptoms among them are more than the general population. Considering these, it is important to explore the organic underpinning of patients presenting with psychosis.

